# Influence of patient motion on quantitative accuracy in cardiac ^15^O-water positron emission tomography

**DOI:** 10.1007/s12350-021-02550-9

**Published:** 2021-03-02

**Authors:** Jonny Nordström, Hendrik J. Harms, Tanja Kero, Jens Sörensen, Mark Lubberink

**Affiliations:** 1 Department of Surgical Sciences/Nuclear Medicine & PET, Uppsala University, Uppsala, Sweden; 2 Centre for Research and Development, Uppsala/Gävleborg County, Gävle, Sweden; 3 Medical Imaging Centre, Uppsala University Hospital, Uppsala, Sweden; 4 Medical Physics, Uppsala University Hospital, Uppsala, Sweden; 5 MedTrace Pharma A/S, Lyngby, Denmark

**Keywords:** Myocardial blood flow, image interpretation, CAD, PET

## Abstract

**Background:**

Patient motion is a common problem during cardiac PET. The purpose of the present study was to investigate to what extent motions influence the quantitative accuracy of cardiac ^15^O-water PET/CT and to develop a method for automated motion detection.

**Method:**

Frequency and magnitude of motion was assessed visually using data from 50 clinical ^15^O-water PET/CT scans. Simulations of 4 types of motions with amplitude of 5 to 20 mm were performed based on data from 10 scans. An automated motion detection algorithm was evaluated on clinical and simulated motion data. MBF and PTF of all simulated scans were compared to the original scan used as reference.

**Results:**

Patient motion was detected in 68% of clinical cases by visual inspection. All observed motions were small with amplitudes less than half the LV wall thickness. A clear pattern of motion influence was seen in the simulations with a decrease of myocardial blood flow (MBF) in the region of myocardium to where the motion was directed. The perfusable tissue fraction (PTF) trended in the opposite direction. Global absolute average deviation of MBF was 3.1% ± 1.8% and 7.3% ± 6.3% for motions with maximum amplitudes of 5 and 20 mm, respectively. Automated motion detection showed a sensitivity of 90% for simulated motions ≥ 10 mm but struggled with the smaller (≤ 5 mm) simulated (sensitivity 45%) and clinical motions (accuracy 48%).

**Conclusion:**

Patient motion can impair the quantitative accuracy of MBF. However, at typically occurring levels of patient motion, effects are similar to or only slightly larger than inter-observer variability, and downstream clinical effects are likely negligible.

**Supplementary Information:**

The online version of this article (10.1007/s12350-021-02550-9) contains supplementary material, which is available to authorized users.

## Introduction

Positron emission tomography (PET) is a useful non-invasive tool for diagnosis and risk stratification of coronary artery disease (CAD).^1^–^3^ Quantification of myocardial blood flow (MBF) has been shown to increase the detection of significant CAD compared to qualitative analysis.^4^–^6^ Cardiac PET is prone to motion artifacts arising from cardiac motion, respiratory motion, cardiac creep, and patient body motion. Patient body motion can occur due to discomfort in response to pharmacological stress, coughing, deep breathing, settling, or gradual relaxation of the thoracic muscles. The choice of stress agent and protocol can impact patient body motion with a more pronounced motion seen using adenosine compared to for regadenoson.^7^,^8^ Patient body motion is a common problem in cardiac PET studies with an occurrence of 30% to 69%, particularly during stress.^7^,^9^–^11^ Cardiac creep, i.e. upward motion of the heart due to decreasing pharmacological stress effect during the scan, has been reported to be a frequent phenomenon (52%) in ^82^Rb PET when hyperemic MBF is induced using regadenoson but has also been reported after treadmill exercise.^12^

Motion challenges the quantitative accuracy in cardiac PET in two ways. First, misalignment between the PET and CT scan leads to erroneous attenuation correction.^13^ Second, misalignment between single PET frames and the segmented left ventricle and blood pool, used for kinetic modeling, leads to inconsistent time-activity curves (TACs) and hence induces errors in the quantification process.

The purpose of the present study was to investigate to what extent motion affects the quantitative accuracy of cardiac ^15^O-water PET/CT and to develop a method for automated motion detection.

For this purpose, motion simulations were performed based on clinical ^15^O-water PET/CT stress scans. In addition, to assess the frequency and magnitude of real occurring motions, a set of clinical scans were visually inspected for motion. A number of different hallmarks of motion detectable in the PET data were identified and the accuracy of automated motion detection based on these hallmarks was evaluated.

## Materials and Methods

### Patients

A total of 64 clinical stress scans from patients referred for assessment of myocardial ischemia with ^15^O-water PET/CT were included in the study. Of these, data from 10 randomly selected patients was used for motion simulations, and motion in the original data was ruled out via visual inspection. Data from 50 different consecutive patients was used for visual assessment of the frequency and magnitude of real occurring clinical patient motion. Finally, data was included from the only four patients (out of circa 500) in our clinic during the last 2 years where severe motion artefacts were noted by the nuclear medicine physician reading the scan. Since only anonymized images were used and the present work was purely an image processing study, this study did not require ethics permission according to the Swedish Law on Medical Research in Humans.

### PET Scanning Protocol

Patients were scanned using a Discovery MI PET/CT (GE Healthcare, Waukesha, WI). The protocol started with a low dose CT scan during normal breathing for attenuation correction. Then 4 min rest and stress scans were perfomed after automated bolus injection of 400 MBq ^15^O-water according to our clinical standard protocol. Hyperemic MBF was induced using a continuous infusion of adenosine (140 μg·(kg × min)^−1^) starting 2 min prior to the start of aquisition and continuing throughout the whole scan.

### Clinical Motion

Evaluation of clinical patient motion was performed by an experienced nuclear medicine physician by visual inspection of 50 consecutive clinical stress scans in aQuant software (MedTrace Pharma AS, Lyngby, Denmark).^14^,^15^ The position of the heart was studied frame by frame in transaxial, coronal, and sagittal plan view. A three point scale was used were a score of zero was defined as no visually existing motion, 1 as motion less than half of the left ventricular (LV) wall thickness, and 2 as motion larger than half of the LV wall thickness.

### Simulations

Simulations of patient motion were performed using an in-house developed tool in Matlab, based on reconstructed image data from 10 clinical stress scans. In addition, a synthetic patient was created to study the effect of motion completely without the influence of existing pathology of the clinical patients. This was done by taking the coordinates from an analyzed original scan from one patient and adding homogenous kinetics to all voxels of RV and LV cavity and myocardial regions as well as extra-cardiac regions.

**Figure 1:**
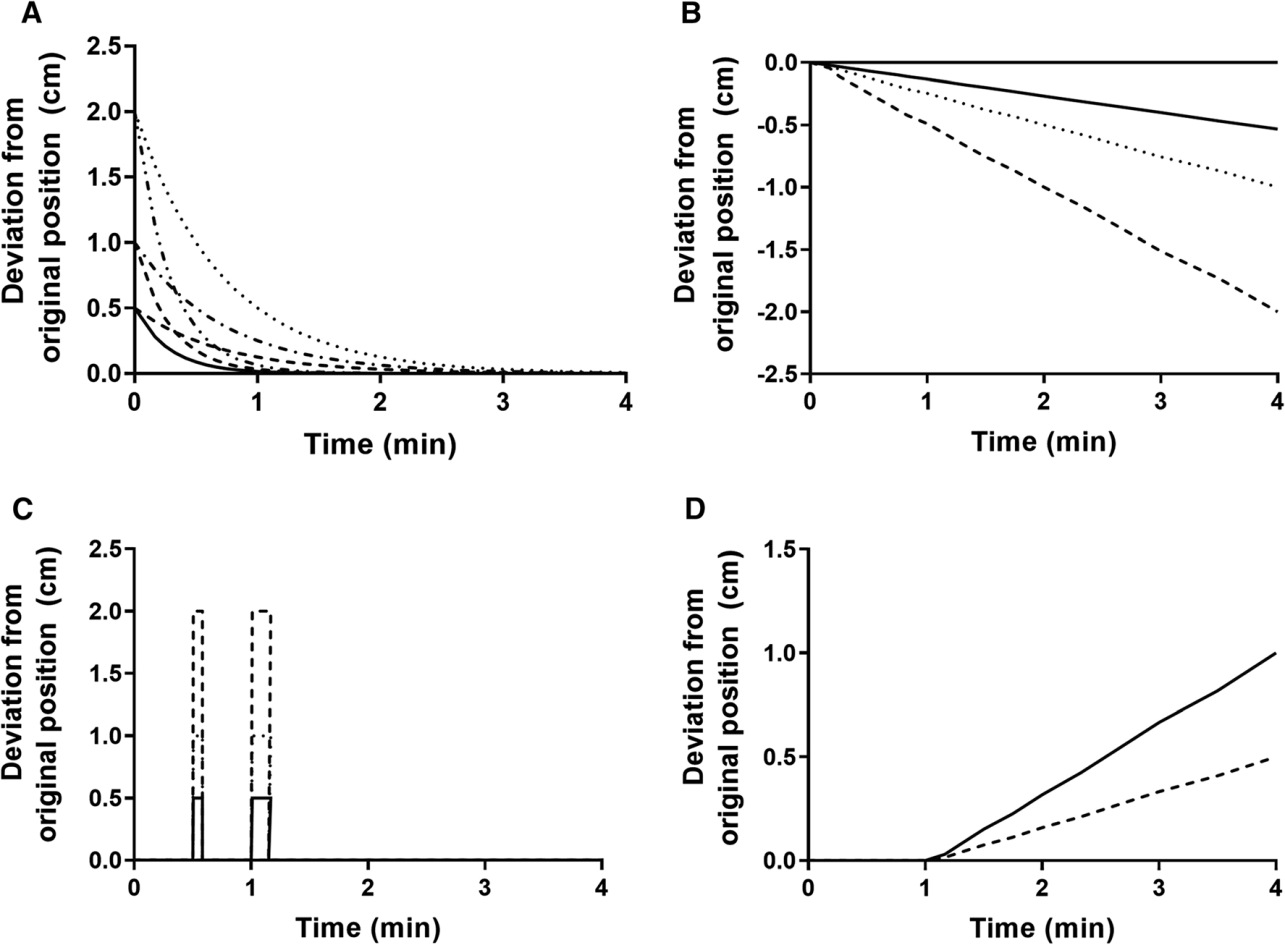
Graphical representation of all simulated motions. (**A**) Type 1 stress agent reaction, (**B**) type 2 caudal linear slide, (**C**) type 3 anterior cough, (**D**) type 4 cardiac creep

Simulations of induced motion were performed by moving the PET matrix frame by frame simulating 17 motions (M1-M17) divided into 4 types at different amplitudes, see Figure 1 and Table 1.

**Table 1:** Description of all 17 simulated motions and percentage of successful automated motion detection

Motion	Simulation	Motion type	Automated detection (%)
1	$0.5 \cdot {\text{e}}^{{ - \frac{\log 2 \cdot t}{0.2}}}$	Type 1: 5 mm stress agent reaction, fast recovery	40
2	$1 \cdot {\text{e}}^{{ - \frac{\log 2 \cdot t}{0.2}}}$	Type 1: 10 mm stress agent reaction, fast recovery	100
3	$2 \cdot {\text{e}}^{{ - \frac{\log 2 \cdot t}{0.2}}}$	Type 1: 20 mm stress agent reaction, fast recovery	90
4	$0.5 \cdot {\text{e}}^{{ - \frac{\log 2 \cdot t}{0.5}}}$	Type 1: 5 mm stress agent reaction, slow recovery	40
5	$1 \cdot {\text{e}}^{{ - \frac{\log 2 \cdot t}{0.5}}}$	Type 1: 10 mm stress agent reaction, slow recovery	100
6	$2 \cdot {\text{e}}^{{ - \frac{\log 2 \cdot t}{0.5}}}$	Type 1: 20 mm stress agent reaction, slow recovery	100
7	5 mm linear slide in caudal direction during whole scan	Type 2: linear slide	40
8	10 mm linear slide in caudal direction during whole scan	Type 2: linear slide	90
9	20 mm linear slide in caudal direction during whole scan	Type 2: linear slide	100
10	5 mm single frame anterior displacement at frame of peak myocardial TAC	Type 3: cough	20
11	10 mm single frame anterior displacement at frame of peak myocardial TAC	Type 3: cough	80
12	20 mm single frame anterior displacement at frame of peak myocardial TAC	Type 3: cough	90
13	5 mm single frame anterior displacement after 1 min	Type 3: cough	30
14	10 mm single frame anterior displacement after 1 min	Type 3: cough	70
15	20 mm single frame anterior displacement after 1 min	Type 3: cough	90
16	5 mm linear slide in cranial direction from 1 min p.i. to end scan	Type 4: cardiac creep	100
17	10 mm linear slide in cranial direction from 1 min p.i. to end scan	Type 4: cardiac creep	80

1. When a stress agent like adenosine is used, discomfort may be experienced leading to a stiffening reaction followed by a gradual relaxation back to original position as the patient gets accustomed. This was simulated as an initial anterior and cranial displacement at the start of the scan with an exponential slide down towards the original position (type 1).
2. Type 2 motion corresponds to a linear slide in caudal direction during the whole scan.
3. A cough was simulated as an anterior motion up and down during a full single frame at the peak of the myocardial TAC (during the first pass through the LV) and after 1 min (type 3).
4. Cardiac creep was simulated as a linear slide in cranial direction starting after 1 min, mimicking the reduced effect of a regadenoson bolus or the effect of terminating an adenosine infusion (type 4).

Effect of misalignment between the CT and PET was included by un-applying the attenuation correction prior to the motion simulations, and then re-applying it after motion was added. This resulted in a simulated movement of the dynamic PET scan with attenuation correction based on the original position. All motion-simulated scans (N = 170) and corresponding 10 original scans were fully analyzed as a complete clinical case in the aQuant software. In short, the software uses a basis function implementation of the single-tissue compartment model with corrections for partial volume and spill-over which was used to compute MBF and perfusable tissue fraction (PTF) images.^16^–^19^ Delineation of the myocardial wall was performed on PTF images, and MBF and PTF were calculated at the global level and for the three coronary artery regions (LAD, RCA, LCX) using non-linear regression of the solution of the single-tissue compartment model.

### Automatic Motion Detection

An algorithm was developed for automatic motion detection that consisted of five different tests (see appendix for a more detailed description):

1. A single point of the myocardial TAC after the first pass in LAD, RCA or LCX with a standardized residual from the modelled fit larger than 3.
2. A significant linear trend in fit residuals in any of the LAD, RCA or LCX regions.
3. A right and left ventricular first pass peak area under the curve (AUC) difference larger than 10%.^20^ This value should be the same irrespective of whether it is based on the left- or right-ventricular TAC, and any differences could indicate motion during the first-pass phase of the scan.
4. A difference of more than 10% between arterial or venous TAC and average myocardial TAC in the last two frames (3 to 4 min). This is based on the fact that ^15^O-water is freely diffusible and hence, the arterial and venous TAC should convergence once equilibrium is reached during the last minute of the scan.
5. A left ventricular spill-over fraction larger than 30% in any myocardial segment according to the 17-segment model. Generally, spill-over fractions are below 20%, and a larger spill-over fraction could be a sign of motion.

The algorithm was applied to all simulated scans (N = 170), the 50 clinical scans that had been visually inspected for existence of motion, and the scans of the 4 patients with known large motion artefacts. Automated motion detection was considered positive if one of the five tests were fulfilled.

### Statistics

Data are presented as mean ± standard deviation (SD). To analyze differences between simulated and original scans, the non-parametric paired Wilcoxon sign-rank test was used. A two-sided *P*-value < .05 was considered significant. Statistical analysis was performed in Matlab.

## Results

### Clinical Motion

Patient motion was detected in 34 of 50 patients (68%) by visual inspection. All observed motions were small with a score of 1. The 4 patients with known motion artefacts were evaluated at a score of 2.

### Simulations

In the original scans, small inter-frame motion was visually observed in 1 of 10 patients, but was judged to not bias the results as it occurred in the last 2 min of the scan and the results of the simulations based on the data of this patient did not stand out in any way. Global stress MBF ranged between 0.83 and 4.3 mL·(g·min)^−1^ for all patients. For one patient, the result from 20 mm cough at peak myocardial TAC (M12) was excluded due to large motion artifacts that made delineation of the myocardial wall impossible.

**Figure 2:**
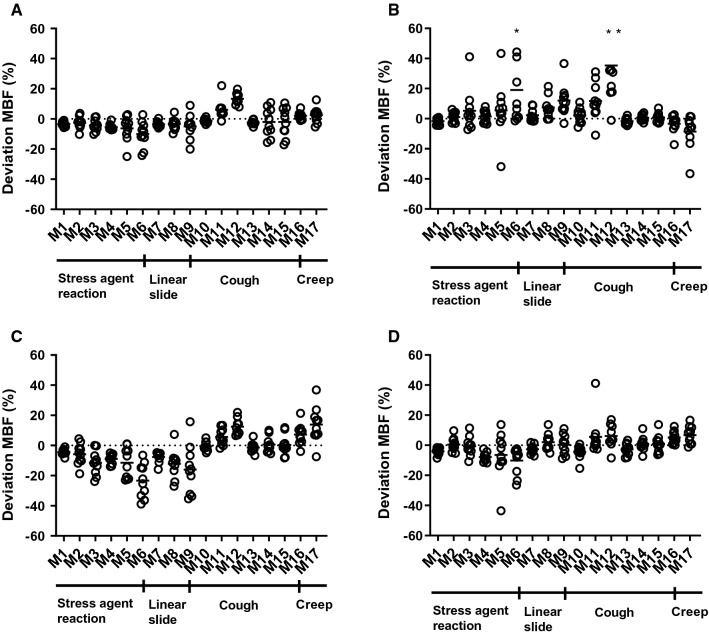
Scatter dot plots showing relative deviation from the original scan of myocardial blood flow (MBF) in all simulated motions (M1-M17) for left ventricle (**A**), left anterior descending artery (**B**), right coronary artery (**C**), and left circumflex (**D**)

**Figure 3:**
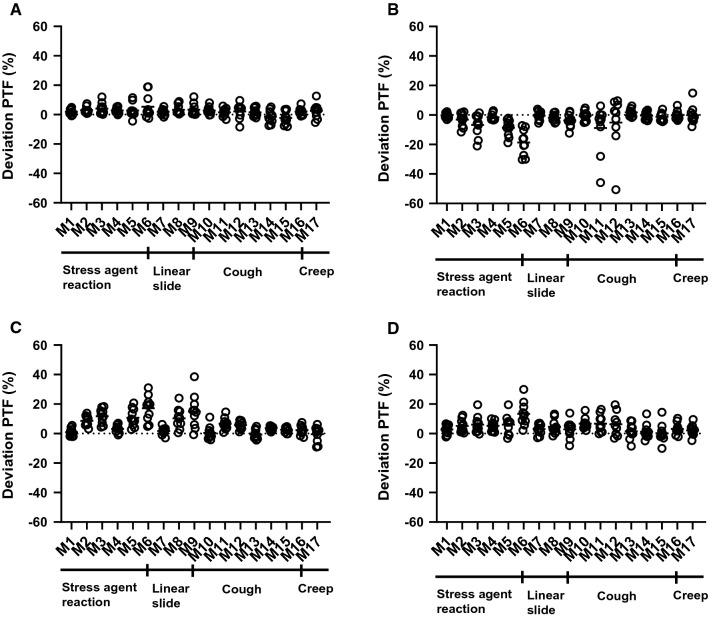
Scatter dot plots showing relative deviation from the original scan of perfusable tissue fraction (PTF) in all simulated motions (M1–M17) for left ventricle (**A**), left anterior descending artery (**B**), right coronary artery (**C**), and left circumflex (**D**)

In Tables 2 and 3, mean relative motion-induced deviations of MBF and PTF from the original values are shown and Figures 2 and 3 shows the corresponding scatter dot plots. Significant deviations from the original scan on the global level were seen in 8 simulated motions for MBF and in 6 simulated motions for PTF. On the global level and across all misalignments, average absolute deviation for MBF was 5.7% ± 4.9% and for PTF 3.2% ± 3.1%. Minor deviations were seen for the smaller motions of 5 mm with an absolute average deviation of 3.1% ± 1.8% compared to 7.3% ± 6.3% for large type of motions at 20 mm. The largest deviation of MBF at 35.3% ± 30.1% was seen in LAD for the 20 mm cough at peak myocardial TAC (M12). For PTF, the largest deviation of -18.6% ± 8.7% was seen in LAD for the 20 mm stress agent reaction with slow recovery (M6).

**Table 2:** Relative mean difference of MBF for each motion compared to the original scan.

Motion	MBF, relative deviation from original scan (%)			
	LV	LAD	RCA	LCX
1	− 3.5 ± 1.5	− 1.6 ± 2.2	− 4.4 ± 2.0	− 4.1 ± 2.2
2	− 2.5 ± 3.9	1.1 ± 3.3	− 5.9 ± 6.9	.5 ± 4.5
3	− 4.3 ± 3.9*	5.2 ± 14.0	− 11.6 ± 7.9**	− .7 ± 6.3
4	− 5.4 ± 2.0	1.4 ± 4.1	− 9.0 ± 3.3	− 7.8 ± 3.1
5	− 6.3 ± 8.2*	5.5 ± 18.4	− 11.5 ± 10.1*	− 6.4 ± 15.7
6	− 10.1 ± 8.4**	19.1 ± 23.0*	− 23.3 ± 11.2**	− 10.1 ± 9.0**
7	− 3.6 ± 1.8	2.2 ± 4.0	− 7.6 ± 3.4	− 2.6 ± 2.9
8	− 3.5 ± 3.9**	7.5 ± 7.0*	− 12.1 ± 9.5**	2.1 ± 5.6
9	− 5.0 ± 7.9*	12.0 ± 10.5*	− 15.9 ± 16.0*	.3 ± 6.7
10	− 1.2 ± 1.5	2.1 ± 5.0	− .5 ± 2.9	− 4.5 ± 4.5
11	6.1 ± 6.2**	11.7 ± 12.2*	5.6 ± 5.5*	5.5 ± 12.9
12	13.3 ± 3.8**	35.3 ± 30.1*	12.5 ± 5.4**	6.1 ± 7.9
13	− 2.5 ± 1.5	− 1.6 ± 2.2	− 1.3 ± 3.3	− 2.7 ± 3.9
14	− 2.1 ± 9.3	.7 ± 2.2	.6 ± 5.2	.9 ± 4.7
15	− 2.0 ± 9.6	.7 ± 3.0	.3 ±6.7	1.0 ± 5.9
16	2.1 ± 2.5*	− 3.3 ± 5.7*	7.3 ± 6.9*	5.2 ± 4.1**
17	2.5 ± 5.0	− 8.6 ± 11.3*	13.8 ± 11.2*	6.8 ± 5.9*

*MBF*, myocardial blood flow; *LV*, left ventricle; *LAD*, left anterior descending; *RCA*, right coronary artery; *LCX*, left circumflex

*P < .05; **P < .005

**Table 3:** Relative mean difference of PTF for each motion compared to the original scan

Motion	PTF, relative deviation from original scan (%)			
	LV	LAD	RCA	LCX
1	2.0 ± 2.0	− .3 ± 1.8	1.0 ± 2.6	2.7 ± 3.2
2	3.0 ± 2.1**	− 3.1 ± 4.8	8.7 ± 3.5**	5.2 ± 4.3**
3	4.1 ± 3.8**	− 6.8 ± 7.5*	11.8 ± 5.1**	6.0 ± 5.9**
4	2.9 ± 2.0	− 1.5 ± 2.3	3.2 ± 2.5	5.3 ± 3.3
5	2.8 ± 4.7*	− 8.8 ± 5.6**	10.8 ± 6.1**	6.1 ± 6.6*
6	5.4 ± 8.0	− 18.6 ± 8.7**	17.1 ± 8.5**	13.4 ± 8.1**
7	1.9 ± 2.1	− .4 ± 3.0	1.8 ± 2.5	3.0 ± 3.6
8	3.2 ± 3.1**	− 1.9 ± 2.6	10.4 ± 6.8**	4.6 ± 4.9*
9	3.4 ± 4.0**	− 3.8 ± 4.6*	15.0 ± 11.3**	2.8 ± 6.0
10	2.8 ± 2.5	− .6 ± 3.4	.4 ± 4.5	6.7 ± 3.9
11	1.5 ± 2.7	− 8.9 ± 15.9	6.6 ± 4.0**	6.7 ± 5.8*
12	2.1 ± 5.3	− 5.1 ± 18.8	5.8 ± 2.9**	6.4 ± 7.8
13	1.6 ± 3.0	1.8 ± 2.6	− .7 ± 3.2	1.6 ± 5.3
14	− 1.5 ± 4.3	− .5 ± 2.6	3.6 ± 1.5*	1.3 ± 5.1
15	− 2.3 ± 4.4	− 1.2 ± 2.8	2.6 ± 1.9*	.3 ± 6.3
16	.5 ± 1.2	− .2 ±3.0	2.3 ± 3.2	3.2 ± 3.8*
17	− 1.9 ± 2.2*	− .1 ±6.1	− .5 ± 5.4	2.2 ± 3.8

*PTF*, perfusable tissue fraction; *LV*, left ventricle; *LAD*, left anterior descending; *RCA*, right coronary artery; *LCX*, Left circumflex

*P < .05; **P < .005

Figures s4 and s5 shows polar plots of the synthetic patient and Figures s6 and s7 polar plots of a typical patient for MBF and PTF with the different types of motion. For motion in the inferior direction (type 1 and 2, M1–9) a typical pattern of a decreased MBF in RCA and increased MBF in LAD was seen. In these cases, PTF showed the opposite effect with an increase in RCA and a decrease in LAD. For cardiac creep (type 4, M16–17) MBF increased in RCA and decreased in LAD, whereas there were only minor effects on PTF. Motion 12, simulating a cough with a large displacement in anterior direction during one frame at peak activity in the myocardial wall, had a large impact on MBF. Later, at 1 min after administration, though, the effect of the same motion was minimal, demonstrating the vulnerability of MBF to sudden motion during the first pass of the tracer.

### Motion Detection

The automatic motion detection algorithm identified motion in 74% of all simulated scans, with a detection rate ranging from 20% to 100% per simulated motion, see Table 1. For motions with 1 to 2 cm maximum amplitudes, the sensitivity of the algorithm was 90%, whereas the sensitivity was 45% for smaller motions with 5 mm amplitudes. In line with the lower detection rate of smaller simulated motions, the clinical motions of score 1 were also hard to detect for the algorithm, with a sensitivity of 53%, specificity of 38%, and accuracy of 48%. However, for the 4 clinical cases where large motions of score 2 had been observed, accuracy of the algorithm was 100%. Average absolute deviations for all detected vs non-detected simulated motions were 10.1% ± 15.0% vs 4.3% ± 4.2% in LAD, 11.0% ± 9.2% vs 5.5% ± 4.5% in RCA, and 6.1% ± 6.8% vs 4.7% ± 3.4% in LCX. All simulated motions with an absolute deviation above 17% in MBF and above 10% in PTF were detected by the algorithm.

## Discussion

Patient motion during cardiac PET studies is a common problem, emphasizing the need for motion correction. In this study, we investigated the influence of patient motion on the quantitative accuracy of ^15^O-water PET. The frequency and magnitude of patient motion in clinical scans was found to be frequent (68%) but generally minor. Simulated motion showed potentially severe consequences on MBF values in motions larger than 10 mm. Using these data, an algorithm for motion detection was developed which detected simulated motions of 10 mm or above with 90% sensitivity, but motion of 5 mm with only 45% sensitivity. As shown by the simulations, MBF was generally decreased in the region of myocardium in which direction the motion occurred. For motions in the inferior direction (type 1 and 2, M1–9) MBF in RCA was decreased, whereas for motions in the cranial direction (type 4, M16–17) MBF in LAD was decreased. PTF trended in the opposite direction, with an increase in the region of myocardium in which direction the motion occurred. This pattern can clearly be seen in the polar plots of the synthetic patient in Figures s4 and s5 for motion types 1, 2 and 4 (M1–9 and M16–17). Type 3 motion with a simulated large cough at the peak of the myocardial TAC (M12) resulted in large artifacts in the polar plot of both MBF and PTF. The vulnerability of MBF to sudden coughing motion during the first pass is clearly demonstrated when almost the entire artifact is gone when the same motion is simulated just after the first pass, at 1 min. However, it should be emphasized that peak first pass occurs during a narrow time window lasting about 15 s, and thus motion artefacts of this type (M12) should be highly unfrequent. In Figures s6 and s7, the same patterns can be seen for a clinical patient, although not as clear as for the synthetic patient.

Even if a clear pattern for each simulated motion type could be seen, the effect of motion is not completely predictable. A high inter-patient variation was seen in the deviations of both MBF and PTF from the original scan, even when the exact same motions were simulated for each patient. For example, the 10 mm linear slide in inferior direction (M8) showed on an overall decrease in MBF for RCA, but for one patient MBF was actually increased. In another patient, MBF in LAD was increased for the 10 mm cardiac creep (M17), whereas this motion overall resulted in a decrease in MBF.

Deviation of both MBF and PTF increased with increasing amplitude of the simulated motion. Global absolute average deviation of MBF was 3.1% ± 1.8% and 7.3% ± 6.3% for motions with maximum amplitudes of 5 and 20 mm, respectively. Koshino et al studied real occurring patient motions in ^15^O-water PET/CT rest scans and observed a deviation for patients with most severe motions of 34% ± 62%, substantially worse than the simulations in the present study.^21^ However, there are differences in the quantification methodology which partly could explain the different results. In the present study which used the aQuant software, parametric images of PTF were used as a reference for delineation of the myocardial wall, which is vulnerable to motion during the first minute of the scan. This is because PTF is basically defined as the uptake rate (*K*_1_) divided by the clearance rate (*k*_2_), which are both to a large extent determined from the earlier part of the scan. For wall delineation in the study by Koshino et al., images consisting of a subtraction of the early time frames from the later frames were used, thus being sensitive to motion during both early and late part of the scan. Koshino et al. also used a second scan with ^15^O-CO as the reference for extracting the LV TAC which might induce additional uncertainties compared to the cluster analysis used in the present study. For other perfusion tracers with a high retention in the myocardium, a sum of the later frames is typically used for wall delineation. Hence, any motion occurring between the early part of the scan, important for perfusion quantification, and the late part of the scan where the wall is delineated, will result in motion-induced errors. Since there is a longer period of time for motion to take place, the likelihood of motion to affect wall delineation increases. On the other hand, for motions extending over a short period of time, for example during a cough, the effect will be more evened out in the longer time frames. Furthermore, for all perfusion tracers except for ^15^O-water, MBF is calculated from the uptake rate K_1_, which is highly dependent on an accurate attenuation correction and errors as high as 500% have been reported in simulations of ^82^Rb scans, which was mainly caused by misalignment with the CT.^8^ For ^15^O-water, with MBF calculated from the washout rate k_2_, it has been shown that PET-CT misalignments have very limited effect on MBF quantification.^13^ Obviously, attenuation correction misalignment errors will instead have significant effects on PTF.

Real occurring clinical interframe patient motion was evaluated by visual inspection, and shown to occur frequently (68%). Importantly, all visually detected motions were small at less than half the LV wall thickness. In line with the presented simulations, this suggests that even if patient motion frequently occurs, the impact on MBF can be expected to be small in the majority of cases. However, the relatively high inter-patient variation in the simulation results implies that, occasionally, even smaller motions at 5 mm amplitude could substantially impact MBF quantification.

In the clinical evaluation of ^15^O-water PET scans it is important to be able to detect the occurrence of motion, in order to identify when a false positive perfusion defect has been induced or when a true one has been masked. Automated motion detection could be used as a beneficial tool to alert the observer to check the scan for motion during analysis. The algorithm used in this study is based on residual analysis to detect inconsistency in TACs, on non-convergence of arterial, venous and late myocardial TACs, or on a large difference in right and left ventricular AUC. Automated motion detection was highly successful at detecting the more severe types of simulated motions, but for motion up to 5 mm the detection rate was low. As all clinical real occurring motions corresponded to maximum one half myocardial wall thickness or roughly 5 mm, automatic motion detection also struggled with the 50 clinical cases. It should be noted, however, that the simulations show that a motion of 5 mm in most cases has an effect that is similar to or only slightly larger than inter-observer variability.^13^ Also, motion-induced errors for simulated motions that the algorithm was not able to detect were small. Hence, these small motions are likely not very problematic from a clinical point of view as they would not have resulted in large errors in MBF. When applied on the 4 patients with known real occurring large motions with a score of 2, accuracy was 100% for the automatic motion detection algorithm. This suggests that the algorithm could be used to alert when severe motion with clinical implications has occurred. The absence of an alert could though not be used to completely rule out a possible impact of clinical importance since the smaller simulated motions occasionally showed a substantial impact on MBF. Reader training will therefore remain a key element in an accurate assessment of motion artifacts for ^15^O-water PET.

In addition to the motion detection algorithm, we could also identify three typical signs of motion that could be used to visually assess motion when viewing parametric images, as shown in Figure s8A–C:

A. A gradient from top left to bottom right in the whole PTF polar plot combined with an unexpected anterior-septal apex PTF defect and an inferior apex defect in MBF. The short axis PTF inferior wall and MBF anterior wall looks smeared and thickened. The PTF defect was not expected for this patient that was evaluated for stable angina without history of infarction. This corresponds to type 1 motion (M6), or relaxation after initial stress agent effects.
B. Substantial smearing of the inferior wall with no distinct separation from the abdominal signal. This is a typical sign of motion in the inferior direction (type 1–2, M1–9).
C. Substantial smearing of the anterior wall. This it is a clear sign of an anterior motion, which could arise during cardiac creep (type 4, M16–17).

Motion detection is an important step towards solving the problem of patient motion but motion correction is the endpoint of the solution. Motion correction can be done either on a frame-by-frame-basis, using image co-registration, or using data-driven methods on raw PET data. The rapid tracer kinetics in cardiac PET, especially during the earlier parts of the scan, results in images with substantially different activity distributions between frames, which highly challenges image co-registration. When using ^15^O-water, motion correction is further challenged since ^15^O-water is freely diffusible and has no retention in the myocardium that otherwise can be used as a reference for image co-registration during later parts of the scan. Promising results of motion correction for dynamic ^82^Rb studies have been published but to our knowledge no motion correction method for ^15^O-water has yet been presented.^19^ In addition, if motion is severe, post-reconstruction motion correction alone is not sufficient since attenuation correction errors are not taken into account. Hence, data-driven motion correction at the list-mode or sinogram level, prior to image reconstruction and provided by scanner vendors, would be preferable.

## Conclusion

Patient motion can impair the quantitative accuracy of ^15^O-water cardiac PET and may induce false positive or false negative results in the most extreme cases. In clinical practice, patient motion is frequently observed but at a magnitude where it has limited effects, similar to or only slightly larger than inter-observer variability. Severe motion with significant impact on MBF could reliably be detected.

## New Knowledge Gained

Patient motion is frequently observed but at a magnitude that has limited impact on the quantitative accuracy of MBF calculated from ^15^O-water PET.

### Supplementary Information

Below is the link to the electronic supplementary material.

Supplementary material 1 (PPTX 406 kb)

Supplementary material 2 (M4A 6698 kb)

Supplementary material 3 (DOCX 1070 kb)

## Appendix

The automated motion detection algorithm was based on 5 different criteria and if one of them were fulfilled the algorithm gave a positive result.

1. Inconsistent time-activity curves (TACs) can be a sign of inter-frame patient motion. A frame point can be considered an outlier if its standardized residual is larger than 3. The standardized residual for a point is defined as the ratio of the point’s residual to the standard deviation of all residuals in the TAC. Each frame point’s residual was calculated as the difference between the measured and fitted activity for that frame. To exclude early frame points where the blood spillover and fast kinetics may induce false positives, only frame points after first pass (defined as all points from peak TAC) were included in the test. If any frame point in any of the 3 coronary regions were above the cut-off value of 3, the test was positive.
2. The residuals between the fit and frame points should be randomly distributed if they are solely caused by noise. If the residuals show a statistically significant trend instead, that is highly suggestive of motion. A linear regression analysis of the residual plot yielding a significant correlation (*P* < 0.05) in any of the three coronary regions gave a positive test.
3. Forward cardiac output (FCO) of the left and right ventricle should be equal. FCO of each ventricle can be calculated via the area under the curve (AUC) of the LV and RV TACs. Large differences in AUCs are not physiological and may therefore be indicative of motion. A threshold of 10% was established from patients in the clinical cohort that had visually been determined to be without motion as their absolute average difference between right and left ventricular AUC plus 2 SD.
4. ^15^O-water is a freely diffusible tracer and hence once equilibrium between blood and myocardium is reached, the activity concentrations in all regions should converge, and lack of convergence is a sign of motion. When the average myocardial TAC in the last two frames (3 to 4 min post injection) is more than 10% different from the arterial or venous blood concentrations at the same timepoints, the test was positive. This threshold was established in the same manner as for test 3 above.
5. As documented for the other PET MPI tracers, a left ventricular blood volume and spill over fraction that is abnormally high could be an indication of early motion during the first pass. Motion during this early phase with subsequent overlap between the blood region and the myocardium will cause a very high spill over fraction. If the left ventricular spill over fraction in any segment of the 17-segment model was above 30% the test was positive. The threshold of 30% was established as in test 3 and 4.

## Abbreviations

MBF: Myocardial blood flow
PTF: Perfusable tissue fraction
PET: Positron emission tomography
CT: Computerized tomography
LAD: Left anterior descending artery
RCA: Right coronary artery
LCX: Left circumflex
LV: Left ventricle
ICC: Intra-class correlation coefficient
