# Supplementary material for: Influence of patient motion on quantitative accuracy in cardiac 15O-water positron emission tomography
Source: J Nucl Cardiol. 2021 Mar 2;29(4):1742–52. doi: 10.1007/s12350-021-02550-9 (PMC9345798; doi:10.1007/s12350-021-02550-9)
Supplement: Supplementary file 1 — Supplementary material 1 (PPTX 406 kb) [file 12350_2021_2550_MOESM1_ESM.pptx]

## Slide 1
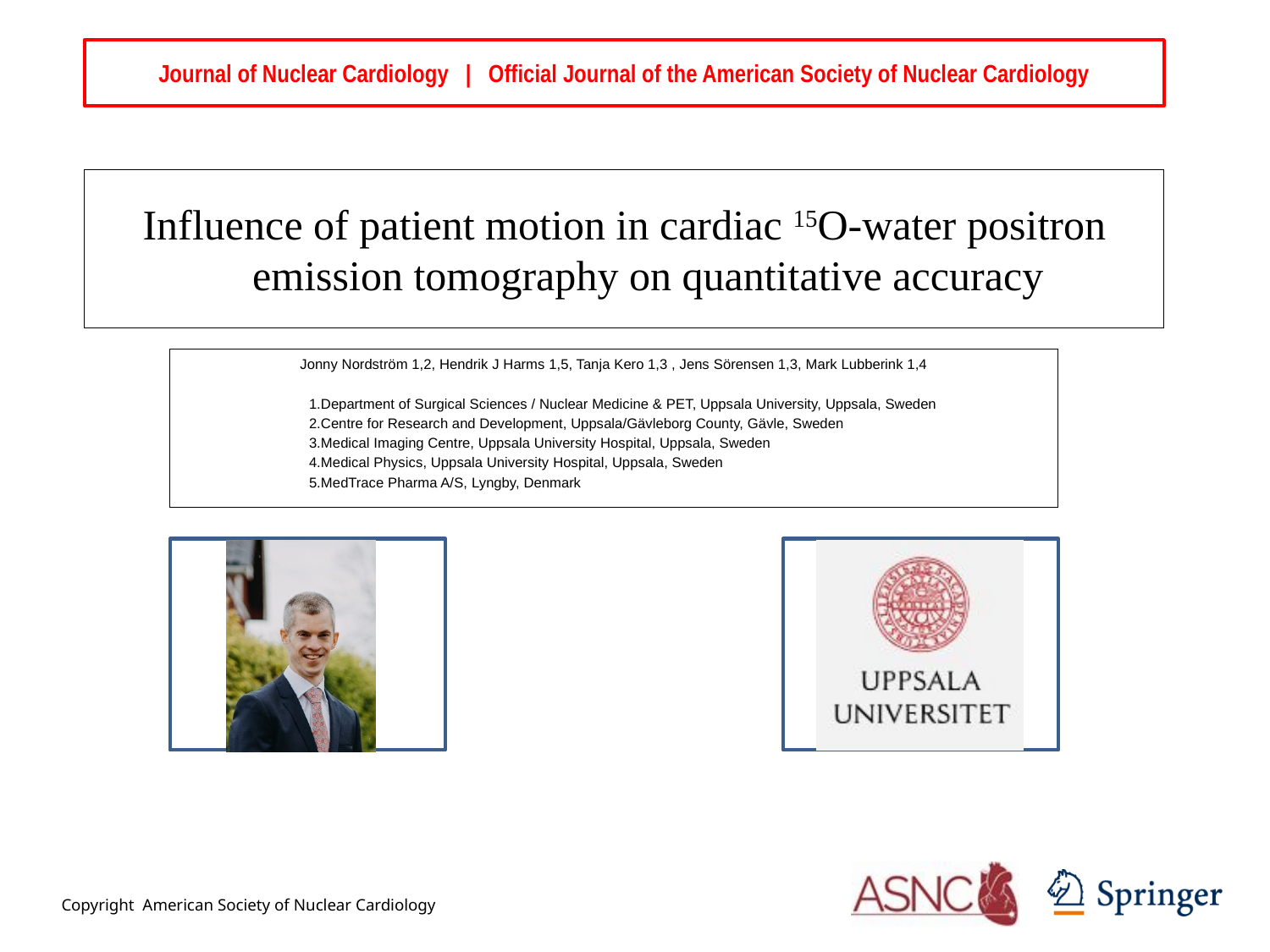

Journal of Nuclear Cardiology | Official Journal of the American Society of Nuclear Cardiology
# Influence of patient motion in cardiac 15O-water positron emission tomography on quantitative accuracy
Jonny Nordström 1,2, Hendrik J Harms 1,5, Tanja Kero 1,3 , Jens Sörensen 1,3, Mark Lubberink 1,4
	1.Department of Surgical Sciences / Nuclear Medicine & PET, Uppsala University, Uppsala, Sweden
	2.Centre for Research and Development, Uppsala/Gävleborg County, Gävle, Sweden
	3.Medical Imaging Centre, Uppsala University Hospital, Uppsala, Sweden
	4.Medical Physics, Uppsala University Hospital, Uppsala, Sweden
	5.MedTrace Pharma A/S, Lyngby, Denmark
Copyright American Society of Nuclear Cardiology

## Slide 2
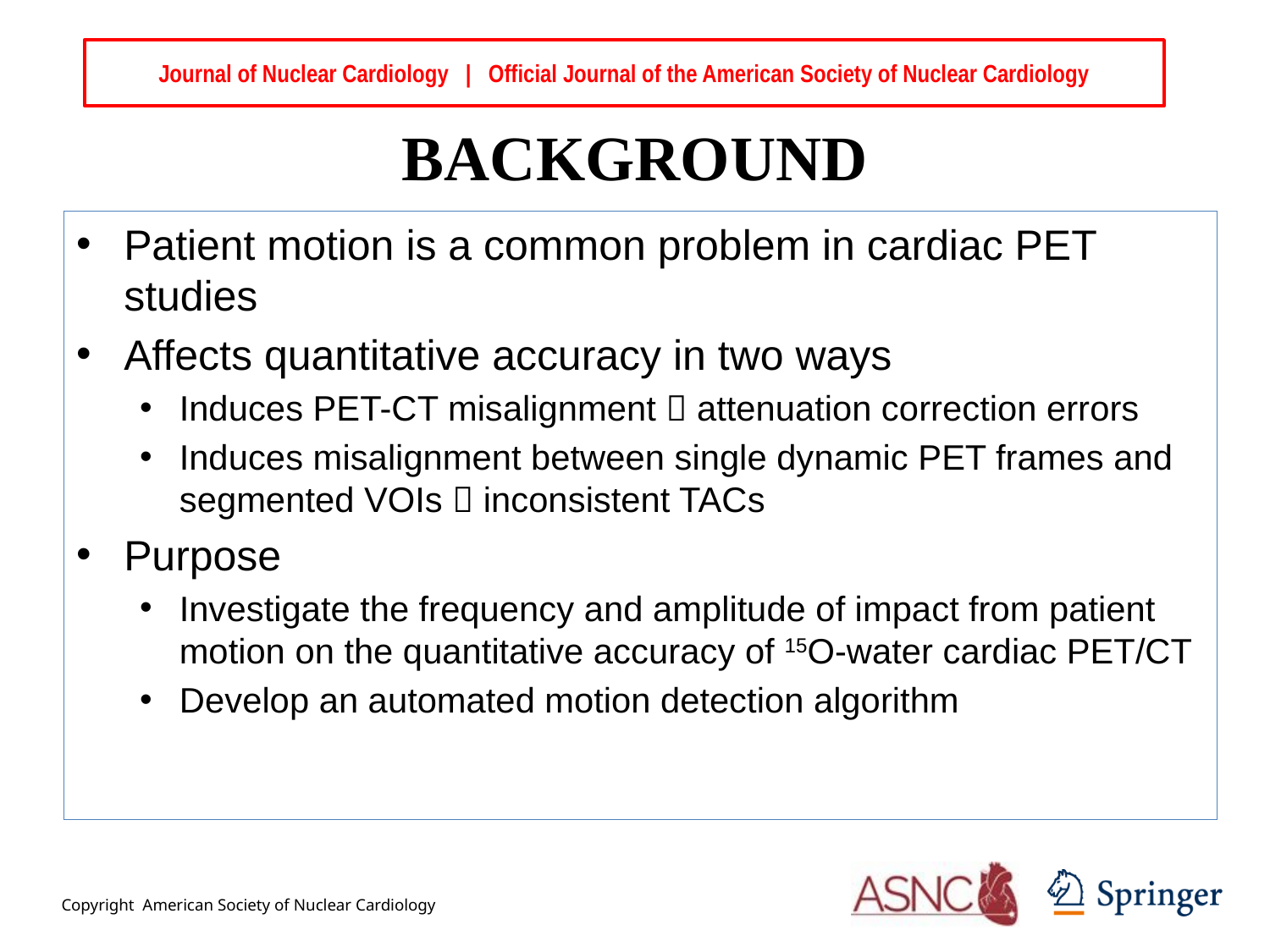

Journal of Nuclear Cardiology | Official Journal of the American Society of Nuclear Cardiology
# BACKGROUND
Patient motion is a common problem in cardiac PET studies
Affects quantitative accuracy in two ways
Induces PET-CT misalignment  attenuation correction errors
Induces misalignment between single dynamic PET frames and segmented VOIs  inconsistent TACs
Purpose
Investigate the frequency and amplitude of impact from patient motion on the quantitative accuracy of 15O-water cardiac PET/CT
Develop an automated motion detection algorithm
Copyright American Society of Nuclear Cardiology

## Slide 3
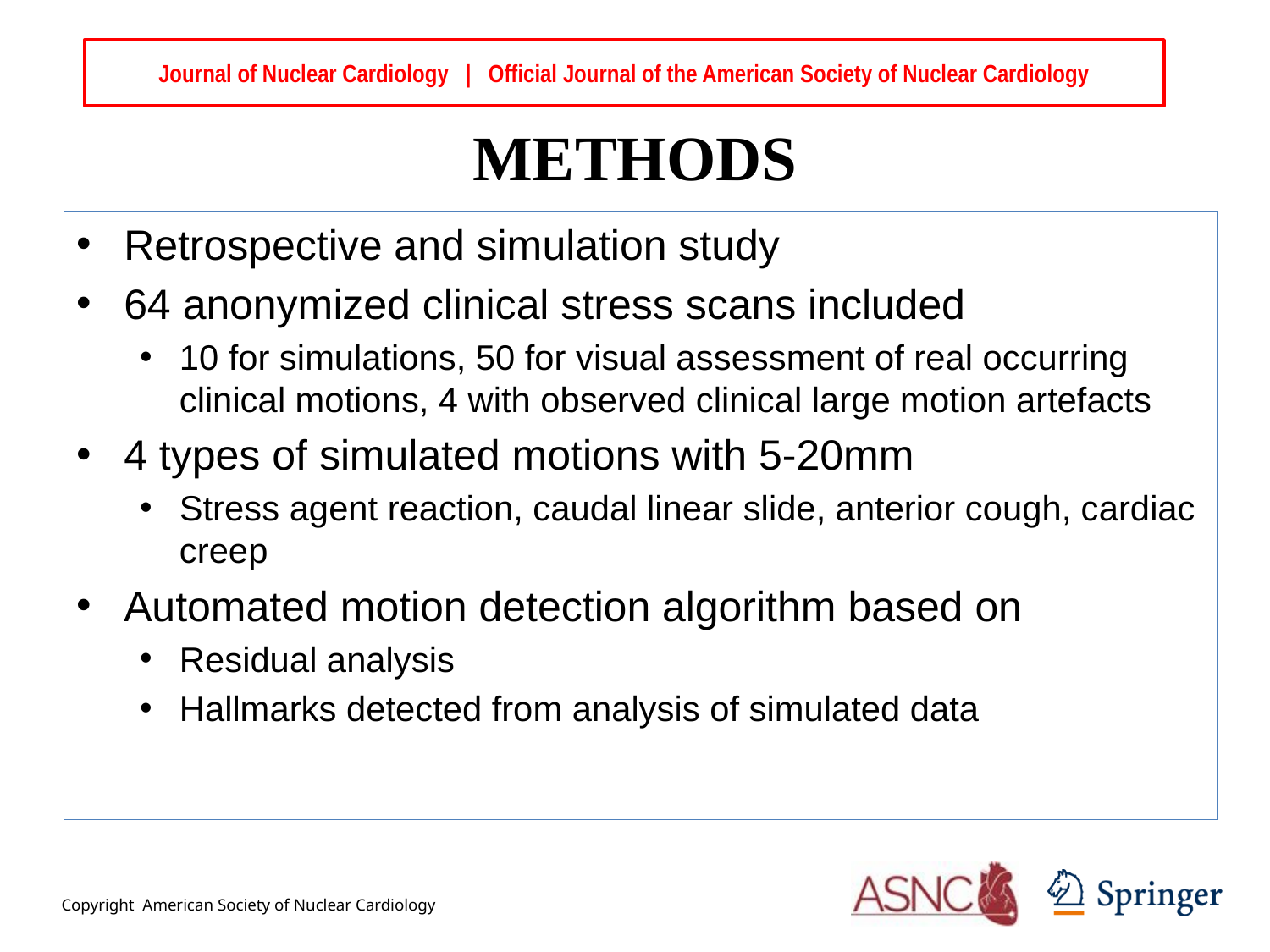

Journal of Nuclear Cardiology | Official Journal of the American Society of Nuclear Cardiology
# METHODS
Retrospective and simulation study
64 anonymized clinical stress scans included
10 for simulations, 50 for visual assessment of real occurring clinical motions, 4 with observed clinical large motion artefacts
4 types of simulated motions with 5-20mm
Stress agent reaction, caudal linear slide, anterior cough, cardiac creep
Automated motion detection algorithm based on
Residual analysis
Hallmarks detected from analysis of simulated data
Copyright American Society of Nuclear Cardiology

## Slide 4
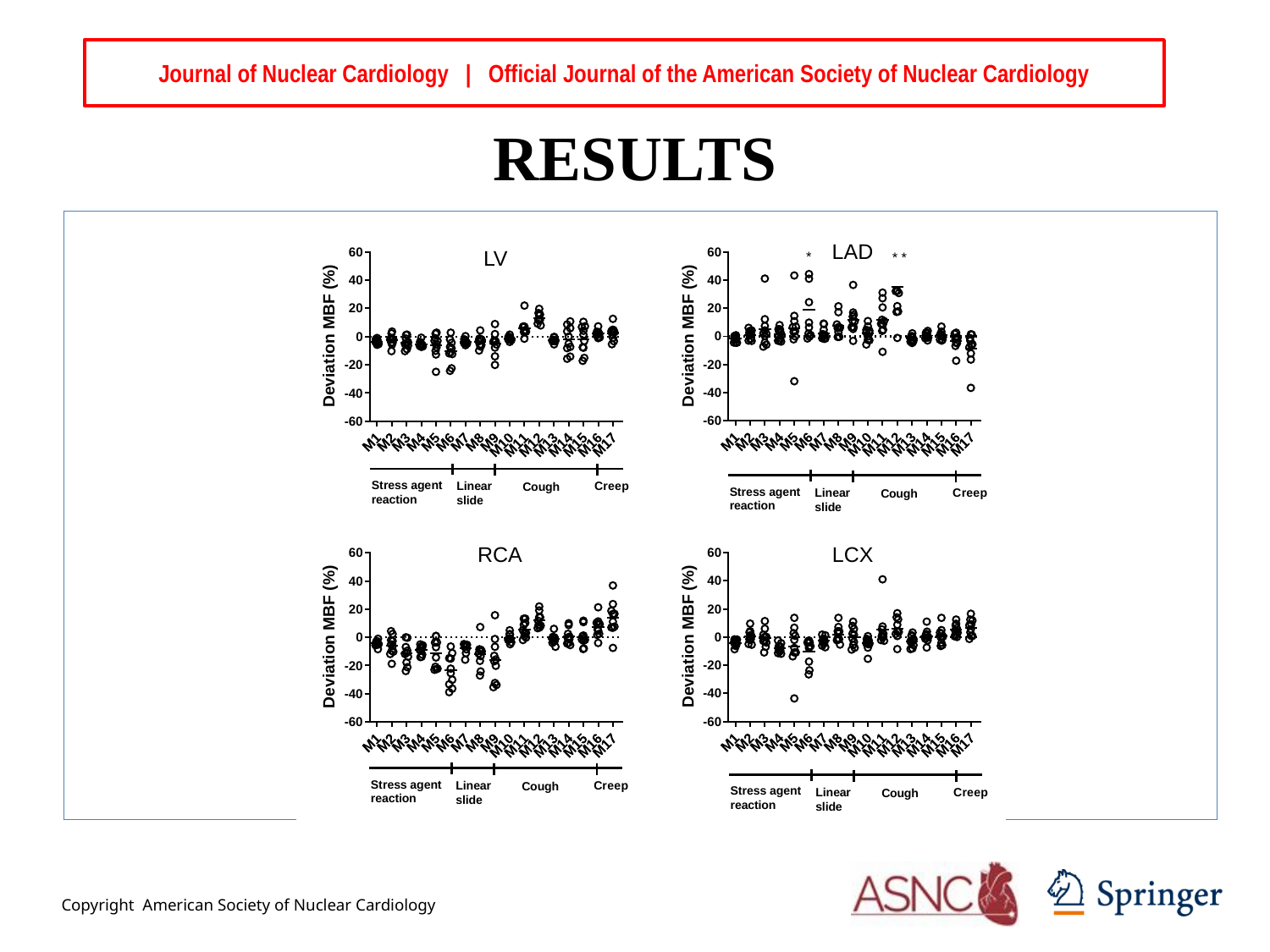

Journal of Nuclear Cardiology | Official Journal of the American Society of Nuclear Cardiology
# RESULTS
LAD
LV
RCA
LCX
Copyright American Society of Nuclear Cardiology

## Slide 5
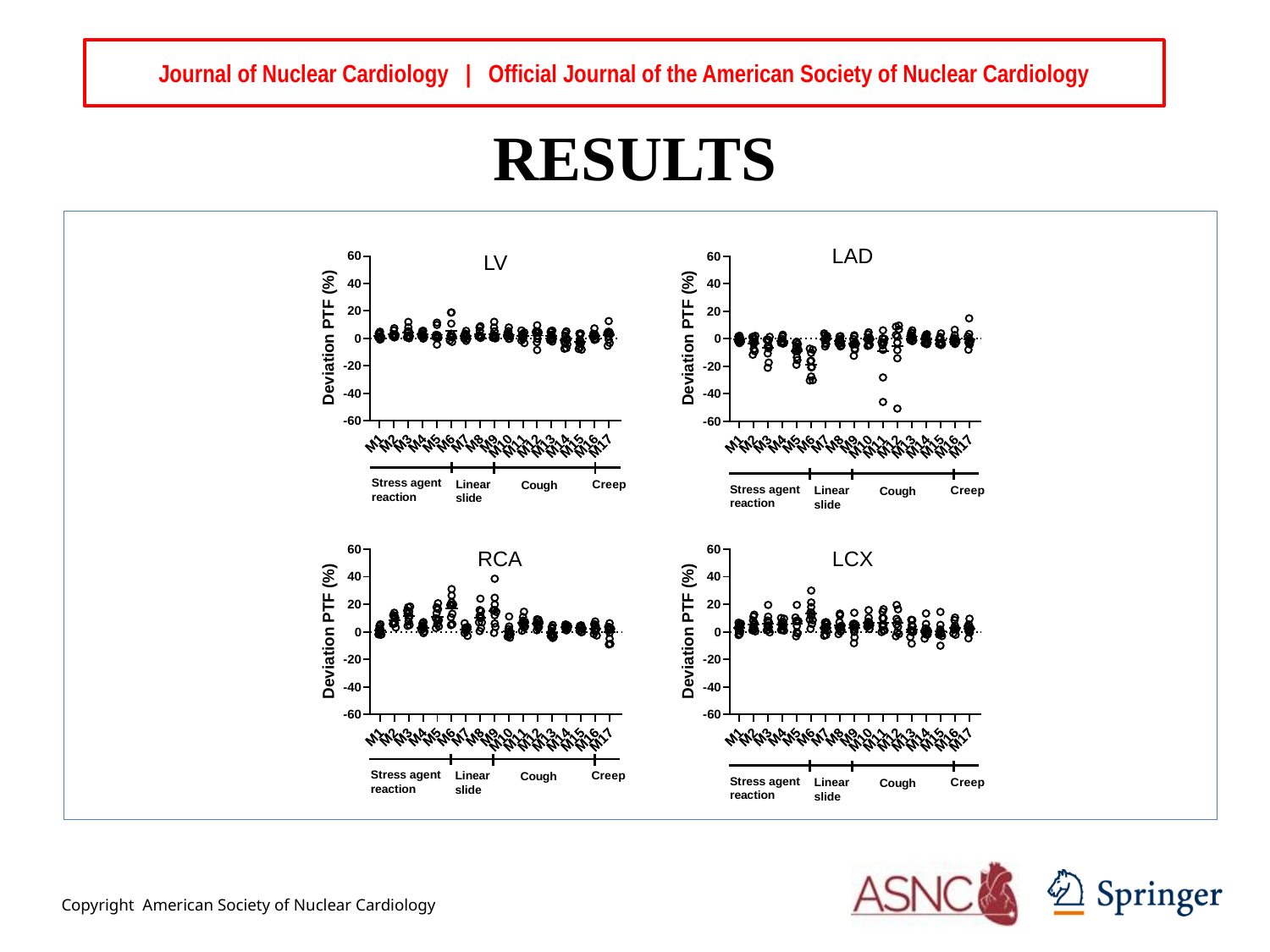

Journal of Nuclear Cardiology | Official Journal of the American Society of Nuclear Cardiology
# RESULTS
LAD
LV
RCA
LCX
Copyright American Society of Nuclear Cardiology

## Slide 6
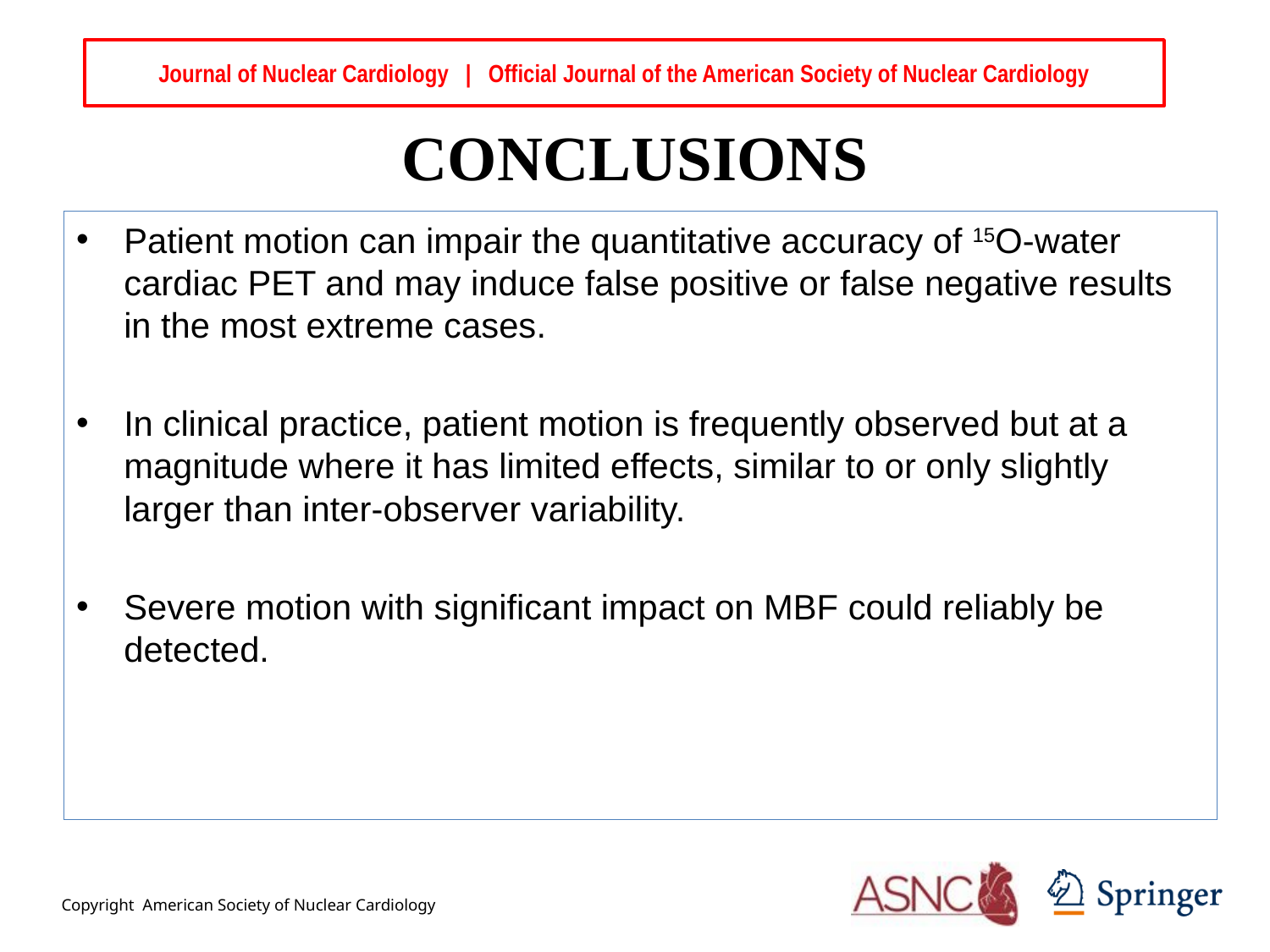

Journal of Nuclear Cardiology | Official Journal of the American Society of Nuclear Cardiology
# CONCLUSIONS
Patient motion can impair the quantitative accuracy of 15O-water cardiac PET and may induce false positive or false negative results in the most extreme cases.
In clinical practice, patient motion is frequently observed but at a magnitude where it has limited effects, similar to or only slightly larger than inter-observer variability.
Severe motion with significant impact on MBF could reliably be detected.
Copyright American Society of Nuclear Cardiology
