# Supplementary material for: Influence of patient motion on quantitative accuracy in cardiac 15O-water positron emission tomography
Source: J Nucl Cardiol. 2021 Mar 2;29(4):1742–52. doi: 10.1007/s12350-021-02550-9 (PMC9345798; doi:10.1007/s12350-021-02550-9)
Supplement: Supplementary file 3 — Supplementary material 3 (DOCX 1070 kb) [file 12350_2021_2550_MOESM3_ESM.docx]

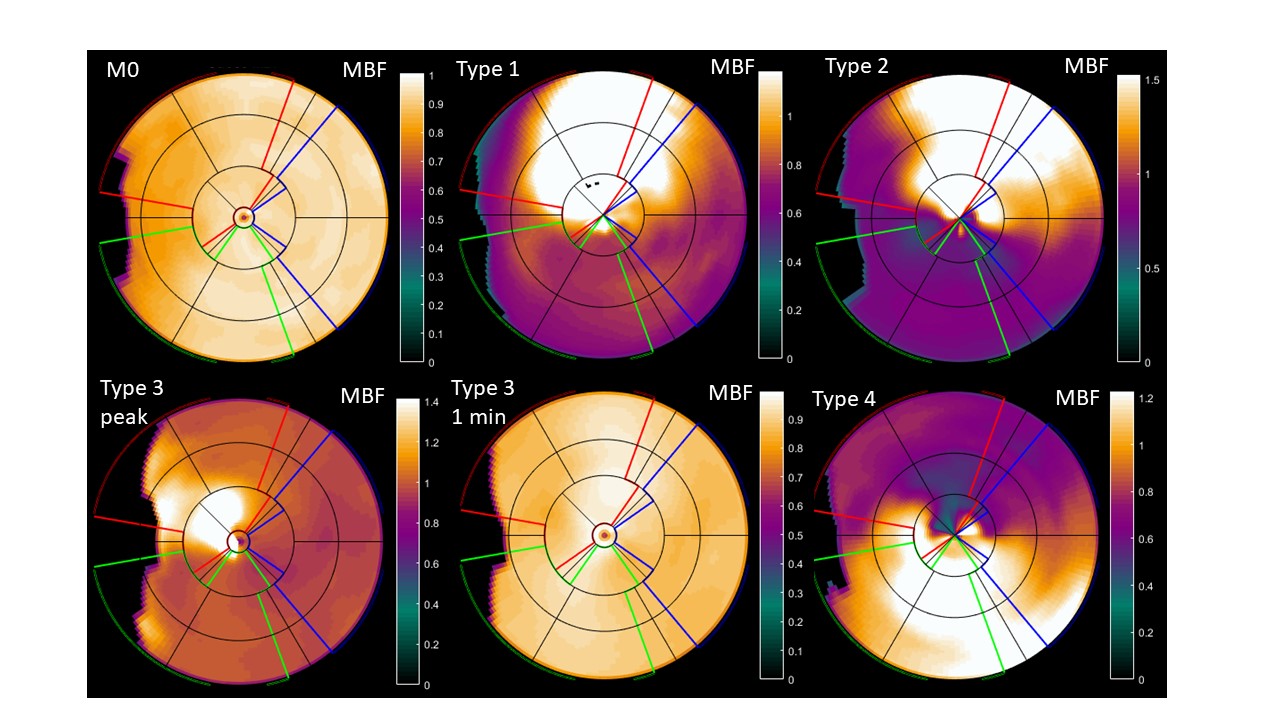


Figure s4 Polar plots showing MBF for the homogenous synthetic patient for all simulated motion types, using the worst case for each type. MBF = Myocardial blood flow; M0 = original scan.


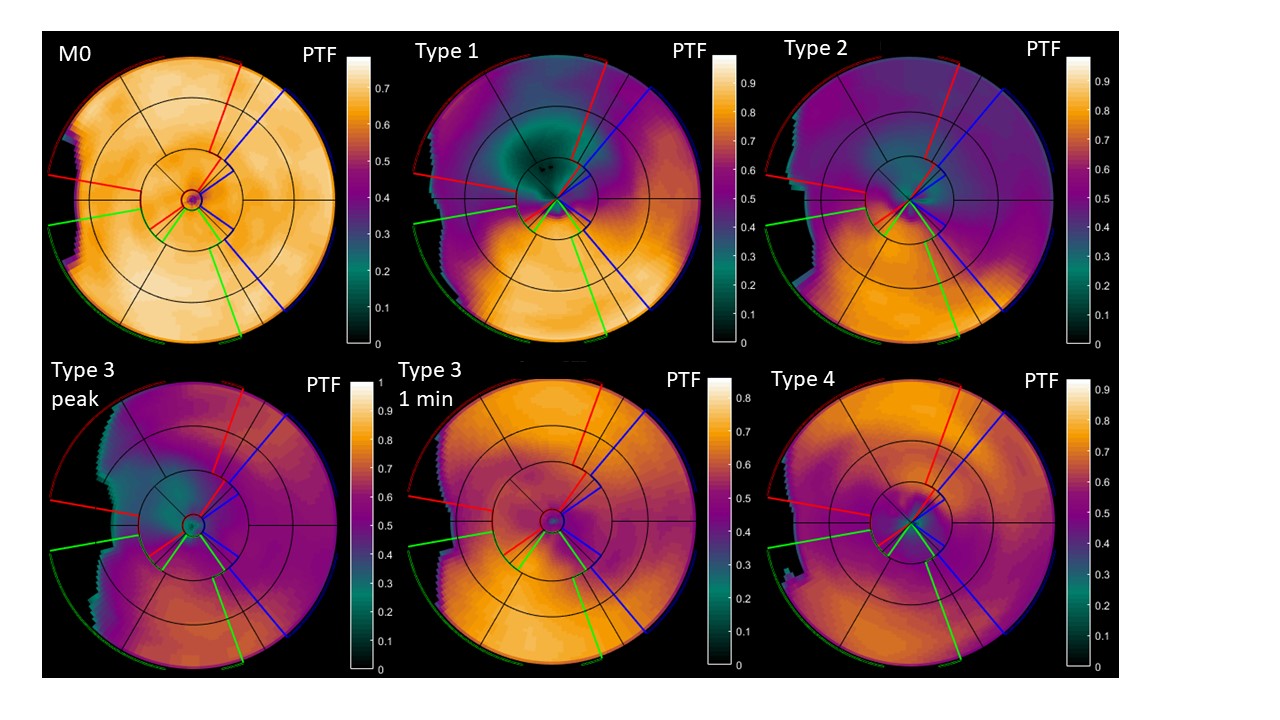

Figure s5 Polar plots showing PTF for the homogenous synthetic patient for all simulated motion types, using the worst case for each type. MBF = Myocardial blood flow; M0 = original scan.


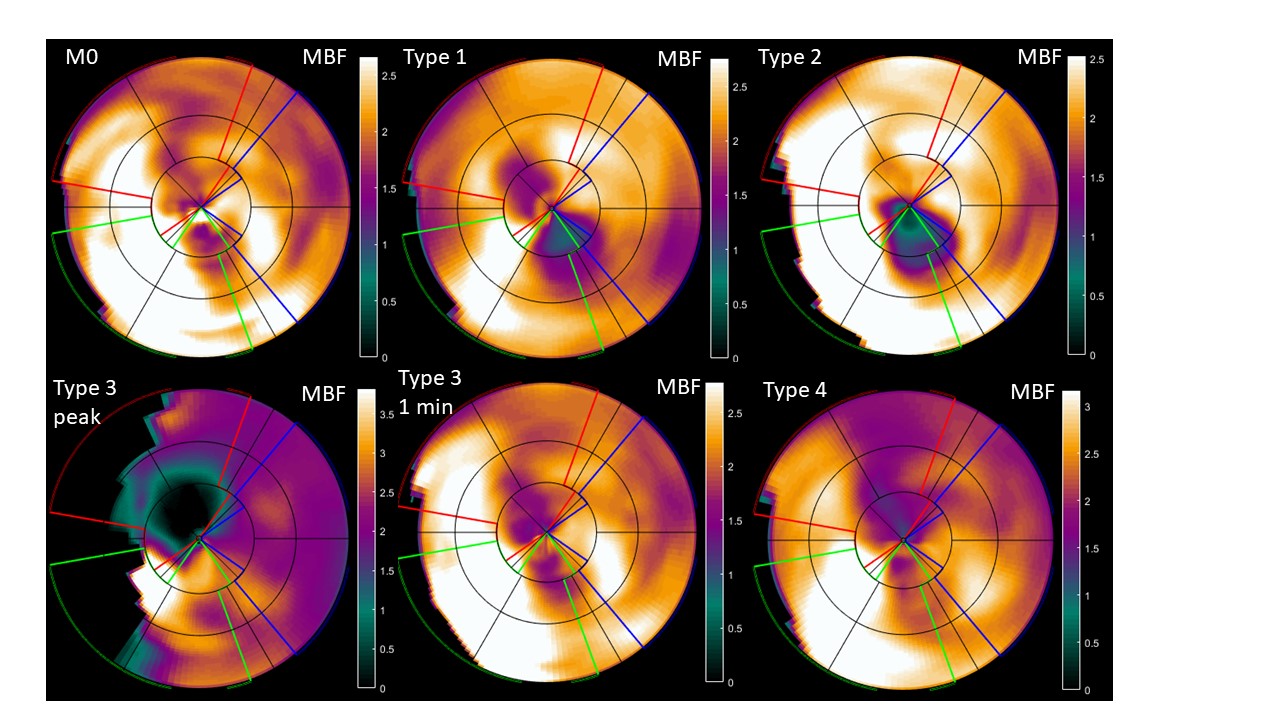


Figure s6 Polar plots showing MBF for one patient for all simulated motion types, using the worst case for each type. MBF = Myocardial blood flow; M0 = original scan.


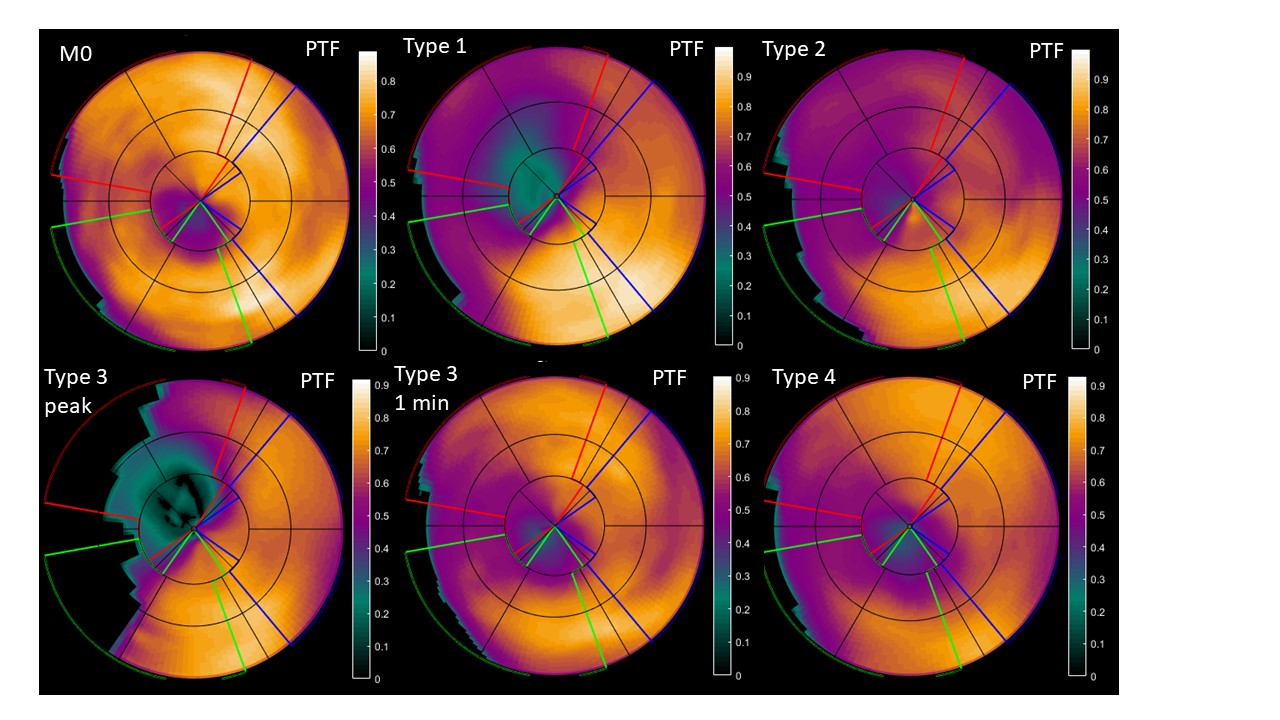


Figure s7 Polar plots showing PTF for one patient for all simulated motion types, using the worst case for each type. PTF = Perfusable tissue fraction; M0 = original scan.


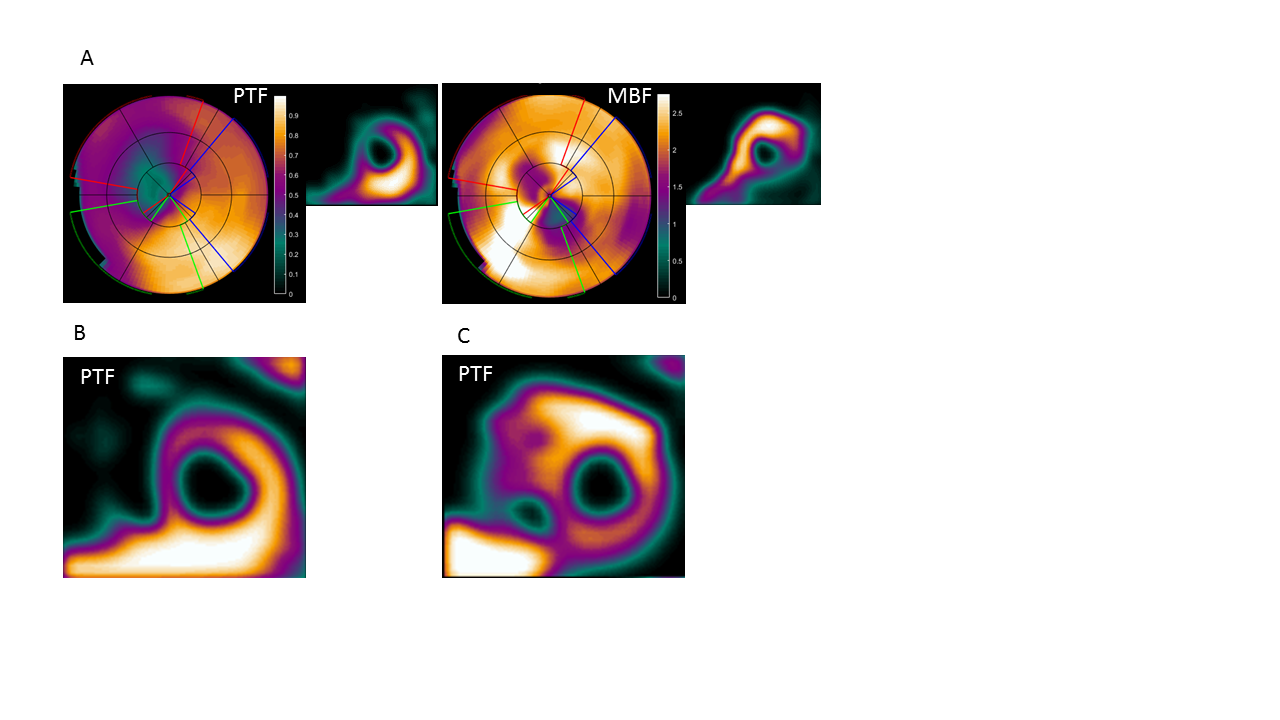
Figure s8 Three signs of motions (A-E) that can be used in the clinical evaluation to visually spot possible motion artifacts. In A, a gradient and a defect is seen in PTF that is not expected for this patient without history of infarction. In B, there is a substantial smearing of the inferior wall which is a sign of inferior motion. In C, there is an obvious smearing of the anterior wall indicating an anterior motion. MBF = Myocardial blood flow; PTF = Perfusable tissue fraction.
